# Cognitive Alexithymia Is Associated with the Degree of Risk for Psychosis

**DOI:** 10.1371/journal.pone.0124803

**Published:** 2015-06-01

**Authors:** Jorien van der Velde, Marte Swart, Sophie van Rijn, Lisette van der Meer, Lex Wunderink, Durk Wiersma, Lydia Krabbendam, Richard Bruggeman, André Aleman

**Affiliations:** 1 Neuroimaging Center, University of Groningen, University Medical Center Groningen, Groningen, The Netherlands; 2 Academy for sociale studies, Hanze University of Apllied Sciences, Groningen, The Netherlands; 3 Lentis, Center for Mental Healthcare, Groningen, The Netherlands; 4 Clinical Child and Adolescent Studies, Leiden University, Leiden, The Netherlands; 5 Department of Psychosis studies, Mental Health Care Friesland, Leeuwarden, The Netherlands; 6 Rob Giel Research Center, University of Groningen, University Medical Center Groningen, Groningen, The Netherlands; 7 Department of Educational Neuroscience, Faculty of Psychology and Education, VU University Amsterdam, Amsterdam, The Netherlands; Leibniz Institute for Neurobiology, GERMANY

## Abstract

Alexithymia is a personality construct denoting emotion processing problems. It has been suggested to encompass two dimensions: a cognitive and affective dimension. The cognitive dimension is characterized by difficulties in identifying, verbalizing and analyzing emotions, while the affective dimension reflects the level of emotional arousal and imagination. Alexithymia has been previously proposed as a risk factor for developing psychosis. More specifically, the two alexithymia dimensions might be differentially related to the vulnerability for psychosis. Therefore, we examined the two dimensions of alexithymia, measured with the BVAQ in 94 siblings of patients with schizophrenia, 52 subjects at ultra-high risk (UHR) for developing psychosis, 38 patients with schizophrenia and 109 healthy controls. The results revealed that siblings and patients had higher levels of cognitive alexithymia compared to controls. In addition, subjects at UHR for psychosis had even higher levels of cognitive alexithymia compared to the siblings. The levels of affective alexithymia in siblings and patients were equal to controls. However, UHR individuals had significantly lower levels of affective alexithymia (i.e. higher levels of emotional arousal and fantasizing) compared to controls. Alexithymia was further related to subclinical levels of negative and depressive symptoms. These findings indicate that alexithymia varies parametrically with the degree of risk for psychosis. More specifically, a type-II alexithymia pattern, with high levels of cognitive alexithymia and normal or low levels of affective alexithymia, might be a vulnerability factor for psychosis.

## Introduction

Alexithymia is a personality construct characterized by difficulties in verbalizing, identifying and analyzing feelings, as well as a restricted fantasy life (fantasizing) and lower emotional arousal (emotionalizing) [1, 2]. Alexithymia is considered to be a risk factor for several psychiatric and neurological disorders such as depression, anxiety, psychosis and somatic disorders [3]. Furthermore, alexithymia is associated with poor social functioning [4] and lower life satisfaction [5].

It has been suggested that alexithymia is not a unitary construct, but that it can be divided into a cognitive and affective dimension [2]. The cognitive dimension refers to the ability to verbalize, identify and analyze feelings, while the affective dimension refers to the level of subjective emotional arousal and the level of fantasizing and daydreaming. Based on these dimensions, different types of alexithymia can be defined [6]. Type-I alexithymia is characterized by high scores on both the cognitive and affective dimension indicating difficulties with the cognitive processing of emotions combined with low levels of emotional arousal and daydreaming. Type-II alexithymia, on the other hand, is characterized by high scores on the cognitive dimension, while scores on the affective dimension are normal or low (i.e. high levels of emotional arousal).

Heightened levels of alexithymia have been reported in schizophrenia [4, 7–12]. More specifically, patients with schizophrenia may show a type-II alexithymia profile, indicating difficulties with identifying, analyzing and verbalizing emotions, while levels of emotional arousal and fantasizing are normal [8] or even heightened [7]. High levels of subjective emotional arousal in the face of a lack of cognitive emotion processing, may have various negative consequences, such as higher levels of negative affect and anxiety [13, 14]. It has further been suggested that alexithymia might be related to psychotic symptoms in patients with schizophrenia [7, 15], however not all studies confirm this association [9, 16].

Previous research suggested that higher levels of cognitive alexithymia might contribute to a greater vulnerability for psychosis [7]. Indeed, some studies have shown subjects at increased risk for developing psychosis to have higher scores on cognitive alexithymia. For example, male siblings of patients with schizophrenia, with an increased genetic risk for developing schizophrenia, showed higher levels of difficulties with verbalizing emotions compared to controls [7]. Furthermore, subjects with an ultra-high risk (UHR) for developing psychosis, appear to have more difficulties in both verbalizing as well as identifying emotions [4].

Siblings of patients with schizophrenia are at increased risk for developing psychosis with one-year transition rates to psychosis between 0.34 and 4.9 percent [17, 18]. Subjects with an UHR for developing psychosis experience subclinical psychotic symptoms and a decline in social and global functioning [19]. These UHR individuals are at even higher risk for developing psychosis with transition rates of 7 to 40 percent after 1 year [20, 21]. If alexithymia indeed contributes to a greater vulnerability for psychosis, one would expect higher alexithymia scores in subjects at UHR for psychosis, compared to siblings. However, to the best of our knowledge, no studies have yet directly compared alexithymia scores between these groups.

The aim of the current study was to examine alexithymia in subjects at genetic risk for psychosis (siblings), subjects at UHR for psychosis and patients with schizophrenia. We hypothesized that the two high-risk groups and the patient group would show a type-II alexithymia pattern. Furthermore, we hypothesized to find a parametric effect of risk on alexithymia scores, with siblings scoring higher compared to controls, UHR individuals scoring higher compared to siblings and patients scoring higher compared to all three groups (controls < siblings < UHR < patients). Finally, we examined whether alexithymia is related to subclinical and clinical psychotic symptoms.

## Material and Methods

### Participants

In the current study we included 109 healthy controls, 94 siblings of patients with schizophrenia, 52 individuals at UHR for developing psychosis and 38 patients with a DSM-IV diagnosis of schizophrenia. The present data were taken from separate studies from our group [4, 22, 23]. There was no overlap between the studies. Furthermore, none of the alexithymia data regarding these studies has previously been published, except for the data from part the UHR group (n = 34 UHR individuals) [4]. The patient, sibling and control samples were independent from earlier alexithymia studies published by our group [7, 8].

All 94 siblings and 57 controls participated in a multi-center (Amsterdam and Groningen) add-on study of the Genetic Risk and Outcome of Psychosis (GROUP) project [24]. The other 52 control subjects were recruited via advertisements. Healthy controls and siblings were excluded if they reported a presence or history of psychiatric or neurological disorders. Sixteen UHR subjects were recruited from the Mental Health Care services in Friesland. Subjects with a 6 or higher on the Prodromal questionnaire (PQ-16 [25]) were assessed with the Comprehensive Assessment of At Risk Mental State (CAARMS [20]) to determine if they met the UHR criteria. The selection procedure was in accordance with the EDIE-NL trial (see [26] for further details). The inclusion procedure of the other 36 UHR subjects was based on the Structured Interview for prodromal symptoms and the Bonn Scale for the Assessment of Basic Symptom-Prediction List and is described in van Rijn et al. (2011) [4]. All the patients with schizophrenia were included from psychiatric institutions across the Netherlands. The clinical diagnosis of the patients was confirmed with the Mini-International Neuropsychiatric Interview (MINI-Plus [27]). Demographic characteristics of the subjects are presented in Table 1.

**Table 1:** Means and standard deviations of demographic variables and alexithymia scores per group and test statistics of group differences.

	HC (n = 109)	Siblings (n = 94)	UHR group (n = 52)	Patients (n = 38)	Test statistic
Demographics					
Gender (% male)	50	46	56	76	χ^2^ = 12.9; p =. 005
Age	31.4 ± 10.4	32.1 ± 8.0	17.8 ± 4.5	34.4 ± 10.6	F_3,289_ = 38.0; p<.001
Education ^a^	6.0 ±. 8	5.9 ±. 8	5.2 ±. 9	5.3 ± 1.1	F_3,289_ = 14.6; p<.001
Alexithymia					
Cognitive dimension	48.8 ± 13.0	55.2 ± 15.8	68.0 ± 16.3	64.6 ± 16.4	F_3,283_ = 14.8; p<.001
Verbalizing	19.7 ± 6.9	21.5 ± 7.2	26.7 ± 7.8	23.8 ± 7.2	F_3,283_ = 6.9; p<.001
Identifying	14.6 ± 4.6	15.5 ± 5.4	22.1 ± 6.7	21.1 ± 5.9	F_3,283_ = 17.4; p<.001
Analyzing	15.9 ± 5.3	17.6 ± 5.7	19.3 ± 6.4	19.7 ± 6.8	F_3,283_ = 3.0; p =. 03
Affective dimension	44.5 ± 11.0	43.4 ± 9.9	39.6 ± 10.0	42.5 ± 10.3	F_3,283_ = 5.3; p =. 001
Fantasizing	22.4 ± 7.2	23.0 ± 7.4	18.8 ± 6.7	21.7 ± 6.6	F_3,283_ = 3.1; p =. 03
Emotionalizing	20.7 ± 4.7	20.4 ± 4.9	20.7 ± 5.1	20.8 ± 6.4	F_3,283_ = 1.1; p =. 35
CAPE scores	HC (n = 66)	Siblings (n = 84)			
Positive symptoms	1.1 ±. 16	1.1 ±. 13	N.A.	N.A.	U = 2389; p =. 07
Negative symptoms	1.4 ±. 35	1.5 ±. 38	N.A.	N.A.	U = 2554; p =. 20
Depressive symptoms	1.4 ±. 36	1.5 ±. 35	N.A.	N.A.	U = 2711; p =. 56
PANSS scores			UHR (n = 49)	Patients (n = 38)	
Positive symptoms	N.A.	N.A.	12.2 ± 3.2	15.3 ± 5.4	U = 628; p =. 009
Negative symptoms	N.A.	N.A.	11.4 ± 3.5	14.4 ± 4.7	U = 544; p =. 001
General symptoms	N.A.	N.A.	26.0 ± 5.4	30.5 ± 8.2	U = 624; p =. 008

^a^ Education according to Verhage (1964)

*Abbreviations*: CAPE: Community Assessment of Psychic Experiences; HC: healthy controls; PANSS: Positive and Negative Syndrome Scale; UHR: Ultra-High Risk

### Ethics statement

All participants gave written informed consent and all studies were approved by either the local medical ethical committee (Medisch Ethische Toetsings Commissie, University Medical Center Groningen or Medical Research ethics committee, University Medical Center Utrecht) or the Mental Healthcare Research Ethics Committee (METIGG, University of Nijmegen). All procedures were carried out according to the declaration of Helsinki.

### Bermond-Vorst Alexithymia Questionnaire

The Bermond-Vorst Alexithymia Questionnaire (BVAQ) is a 40-item self-report scale used to assess alexithymia. The BVAQ consists of five subscales (eight items per scale), identifying, verbalizing, analyzing, emotionalizing and fantasizing as defined by Nemiah and Sifneos [28]. Participants rated on a 5-point Likert scale to what extent the statements applied to them (1 = certainly does not apply to me, 5 = certainly does apply to me). Higher scores on the BVAQ indicate more pronounced alexithymic characteristics. Previous studies have confirmed the five-factor structure of the BVAQ and have shown that the BVAQ has good psychometric properties [2, 29].

Using the BVAQ, a second-order distinction can be made in which the factors emotionalizing and fantasizing are grouped into the affective dimension, and the subscales identifying, verbalizing, and analyzing feelings into the cognitive dimension of alexithymia. The validity of this two-factor structure has been demonstrated and confirmed by seven factor-analyses in several populations [6, 30, 31], however not all studies have replicated this [30, 32].Therefore, in the current study, both the two alexithymia dimensions as well as the five subscales were used to examine alexithymia.

### Community Assessment of Psychic Experiences

The community assessment of psychic experiences (CAPE) is a 42-item self-report questionnaire, which was applied to examine self-reported psychotic-like experiences in the controls and siblings [33]. The frequency of positive, negative and depressive symptoms was measured on a 4-point scale (1 = never; 4 = nearly always). The average score per subscale (the frequency of positive, negative and depressive symptoms) was used in these analyses.

### Positive and negative syndrome scale

To examine the clinical characteristics of the UHR individuals and patients with schizophrenia, the semi-structured interview Positive and Negative Syndrome Scale (PANSS [34]) was administered. In this 30-item interview, positive, negative and general symptoms of psychosis that occurred in the past week were measured.

### Statistical analyses

To examine possible differences between the groups on demographic variables, two analyses of variance (ANOVA) were performed with age and education as dependent variables and group (controls, siblings, UHR, patients) as an independent variable. Furthermore, a chi-square test was performed to examine gender differences between groups. For both analyses the statistical significance level was set at *p*<.05. The results revealed significant group differences on demographic variables (see Table 1). Therefore, these were included in further analyses. In addition, the two UHR groups were included through different inclusion criteria. Therefore, the UHR group from the study of Van Rijn et al. (2011)[4] was compared with the sample from Groningen on age, education, alexithymia scores and PANSS scores using two-sample t-tests. Furthermore, a chi-square test was performed to examine gender differences between the groups.

To examine group differences on the alexithymia dimensions, two ANCOVA’s were performed. The first analysis contained the cognitive alexithymia dimension as a dependent variable and group and sex as independent variables. The second analysis, contained the affective alexithymia dimension as a dependent variable. Age and education were included as covariates in both ANCOVA’s. If the ANCOVA’s resulted in a significant main effect (*p*<.05), post-hoc comparisons, comparing the groups to each other, were performed. The significance level for these post-hoc tests was set at *p*<.05, corrected for multiple comparisons applying a Bonferroni correction.

To examine whether possible differences were driven by specific subscales, two MANCOVA’s were performed. In the first analysis, the cognitive BVAQ subscales (i.e. identifying, verbalizing, analyzing) were included as dependent variables and gender and group were included as independent variables (3x4x2 design). In the second MANCOVA, the affective BVAQ subscales (i.e. emotionalizing and fantasizing) were included as dependent variables resulting in a 2x4x2 design. In both MANCOVA’s, age and level of education were included as covariates. The main effects of gender and group on alexithymia, as well as the interaction between the two were examined at a significance level of *p*<.05. Only if the MANCOVA’s resulted in a significant multivariate effect, univariate group effects were examined. For all univariate tests which showed a significant effect of group on alexithymia scores (*p*<.05), post-hoc comparisons were performed, comparing the groups to each other. The significance levels of the post-hoc tests were corrected for multiple testing using a Bonferroni correction. All abovementioned analyses were also repeated without including sex, education and age to examine the effects of the included covariates on the findings. Furthermore, previous research has suggested that assessing alexithymia in adolescents through the TAS-20 might not be reliable [35, 36]. As far as we know, the reliability of the BVAQ has not yet been investigated in adolescents. To make sure possible reliability issues in the adolescent group did not affect the current findings, the abovementioned analyses were also performed excluding subjects below the age of 18.

To examine the correlation between alexithymia and CAPE or PANSS scores, Spearman’s rho correlations were performed. Non-parametric testing was chosen because the CAPE scores and the PANSS scores were not normally distributed. For the controls and siblings, correlations between the two alexithymia dimensions and positive, negative and depressive CAPE scores were examined. The significance level was set at *p*<.008 to correct for multiple comparisons (Bonferroni correction on 6 tests). In the UHR individuals and schizophrenia patients, the correlations between the two alexithymia dimensions and the positive, negative and general PANSS scores were examined. The significance level was set at *p*<.008 to correct for multiple comparisons (Bonferroni correction on 6 tests).

## Results

### Demographic data

The results revealed a significant main effect of group on age and education (see Table 1). Furthermore, the groups differed significantly on gender (see Table 1). Therefore, age, education and gender were included in all further analyses. The two UHR samples differed significantly on age (M_vanRijn_ = 15.6; M_Groningen_ = 22.9; t = 6.4; p<.001) and the verbalizing alexithymia scale (M_vanRijn_ = 24.9; M_Groningen_ = 30.7; t = 2.5; p =. 02). No other significant differences between the two UHR samples were found (lowest p =. 18).

### The cognitive and affective alexithymia dimension

The alexithymia data were checked for outliers (>3 s.d.), no outliers were found on the cognitive dimension, the affective dimension or the alexithymia subscales in any of the groups. Correlation analyses revealed significant associations between the three cognitive subscales (verbalizing—identifying: *r* =. 56, *p*<.001; verbalizing—analyzing: *r* =. 53, *p*<.001; identifying—analyzing: *r* =. 44, *p*<.001) and the two affective subscales (fantasizing—emotionalizing: *r* =. 24, *p*<.001), confirming the two-facture structure proposed by Vorst and Bermond (2001) [2]. Furthermore, the cognitive and affective alexithymia dimensions were only weakly correlated to each other (*r* =. 13, *p* =. 02).

The results showed a significant main effect of group on the cognitive alexithymia dimension (see Table 1, partial η^2^ =. 14). Furthermore, a significant main effect of gender (F_1,283_ = 15.1, *p*<.001, partial η^2^ =. 05) on the cognitive dimension was found (i.e. men had higher scores than women), while the main effect of age (F_1,283_ =. 30, *p* =. 59, partial η^2^ =. 001), education (F_1,283_ = 2.5, *p* =. 12, partial η^2^ =. 009) and the group*gender interaction (F_3,283_ = 1.3, *p* =. 26, partial η^2^ =. 01) were not significant. Post-hoc comparisons revealed that all groups (siblings, UHR and patients) had significantly higher cognitive alexithymia scores compared to controls (see Table 2). Furthermore, the UHR group had higher cognitive alexithymia scores than siblings, while the patients with schizophrenia did not significantly differ from the siblings or the UHR group (see Table 2). Repeating the analyses without including gender, age and education revealed the same significant main effect of group (see Table A in S1 File). The post-hoc tests without including covariates also revealed the same group differences, except that the difference between patients and siblings was now also significant (see Table B in S1 File).

**Table 2:** Post-hoc results (mean difference and *p*-value) of group differences on the cognitive and affective alexithymia dimension.

		Cognitive dimension	Affective dimension
HC	Siblings	-7.0; *p* =. 004*	.9; *p* = 1.0
	UHR	-17.1; *p*<.001*	7.7; *p* =. 001*
	Patients	-12.9; *p*<.001*	3.6; *p* =. 59
Siblings	UHR	-10.1; *p* =. 004*	6.7; *p* =. 007*
	Patients	-5.9; *p* =. 39	2.7; *p* = 1.0
UHR	Patients	-4.2; *p* = 1.0	-4.0; *p* =. 75

* Significant at *p*<.05, corrected for multiple comparisons applying a Bonferroni correction

*Abbreviations*: HC: healthy controls; UHR: Ultra-High Risk

For the affective alexithymia dimension, the results revealed a significant main effect of group (see Table 1, partial η^2^ =. 05) and a significant main effect of education (i.e. individuals with higher levels of education had lower levels of affective alexithymia) (F_1,283_ = 17.4, *p*<.001, partial η^2^ =. 06). The main effects of age (F_1,283_ =. 06, *p* =. 80, partial η^2^<.001), gender (F_1,283_ = 2.7, *p* =. 10, partial η^2^ =. 009) and the group*gender interaction (F_3,283_ =. 61, *p* =. 61, partial η^2^ =. 009) were not significant. Post-hoc comparisons revealed that the UHR group had significantly lower scores on the affective dimension compared to controls and siblings (see Table 2). No other group differences on this dimension were found (see Table 2). The analyses without including covariates revealed the same group differences (see Tables A and B in S1 File).

The abovementioned analyses were also performed excluding individuals below 18 years old. These analyses revealed the same group differences (see Tables A-C in S2 File).

### The alexithymia subscales

To examine whether specific subscales were underlying the group differences on the two alexithymia dimensions, two MANCOVA’s were performed. The results of the first MANCOVA showed a main effect of group on the cognitive alexithymia subscales (F_9,849_ = 6.4; *p*<.001; Pillai’s Trace =. 19; partial η^2^ =. 06). Furthermore, a significant main effect of education (i.e. individuals with higher levels of education had lower scores on the cognitive alexithymia subscales) (F_3,281_ = 4.0; *p* =. 009; Pillai’s Trace =. 04; partial η^2^ =. 04) and gender (i.e. men had higher scores than women) (F_3,281_ = 5.5; *p* =. 001; Pillai’s Trace =. 06; partial η^2^ =. 06) were found, while the main effect of age (F_3,281_ = 1.1; *p* =. 37; Pillai’s Trace =. 01; partial η^2^ =. 01) and the group*gender interaction (F_9,849_ = 1.3; *p* =. 22; Pillai’s Trace =. 04; partial η^2^ =. 01) were not significant. Follow-up analyses revealed that the groups differed significantly on all three subscales of the cognitive alexithymia dimension (i.e. verbalizing, identifying and analyzing) (see Table 1). Post-hoc comparisons are presented in Table 3 and show that UHR individuals differed significantly from controls and siblings on the verbalizing scale. Regarding identifying, the UHR and patient group both differed significantly from controls and siblings, but not from each other. On the analyzing scale, the only marginally significant difference was found between the controls and the siblings. The analyses without including covariates revealed nearly the same pattern, except in these analyses patients and UHR individuals also differed significantly from controls on the analyzing subscale (see Tables A and C in S1 File).

**Table 3:** Post-hoc results (mean difference and *p*-value) of group differences on the cognitive alexithymia subscales.

		Verbalizing	Identifying	Analyzing
HC	Siblings	-2.1; *p* =. 22	-1.0; *p* = 1.0	-1.9; *p* =. 09
	UHR	-6.1; *p*<.001*	-6.7; *p*<.001*	-1.8; *p* =. 61
	Patients	-3.4; *p* =. 16	-5.5; *p*<.001*	-2.7; *p* =. 16
Siblings	UHR	-4.0; *p* =. 03*	-5.7; *p*<.001*	.1; *p* = 1.0
	Patients	-1.3; *p* = 1.0	-4.5; *p* =. 001*	-.8; *p* = 1.0
UHR	Patients	2.7; *p* =. 86	1.2; *p* = 1.0	-.9; *p* = 1.0

* Significant at *p*<.05, corrected for multiple comparisons applying a Bonferroni correction

*Abbreviations*: HC: healthy controls; UHR: Ultra-High Risk

The second MANCOVA on the two subscales of the affective alexithymia dimension, revealed no significant effect of group (F_6,566_ = 1.7; *p* =. 12; Pillai’s Trace =. 04; partial η^2^ =. 02). Furthermore, the main effects of age (F_2,282_ = 3.8; *p* =. 03; Pillai’s Trace =. 03; partial η^2^ =. 03), education (F_2,282_ = 8.2; *p*<.001; Pillai’s Trace =. 06; partial η^2^ =. 06) and gender (F_2,282_ = 25.7; *p*<.001; Pillai’s Trace =. 15; partial η^2^ =. 15) were significant (i.e. males with lower levels of education and higher age had higher levels on the affective dimension subscales), while no significant group*gender interaction (F_6,566_ = 1.5; *p* =. 18; Pillai’s Trace =. 03; partial η^2^ =. 02) was found. Performing the analysis without including gender, age and education did reveal a significant main effect of group on the affective dimension (F_6,578_ = 2.3, *p* =. 03; Pillai’s Trace =. 05; partial η^2^ =. 02). Follow-up analyses revealed a significant effect of group on fantasizing, but not emotionalizing (see Table A in S1 File). Post-hoc comparisons showed that only the UHR group had significantly lower scores on the fantasizing subscale compared to controls and siblings (see Table C in S1 File).

The abovementioned analyses were also performed excluding individuals below 18 years old. These analyses revealed the same group differences (see Tables A-C in S2 File).

### Correlations between alexithymia and psychopathology

The CAPE was only administered in a subsample of 87 siblings and 66 controls (total n = 153, for mean scores see Table 1). PANSS data were missing for 3 UHR individuals, which resulted in a total sample of 87 subjects (49 UHR individuals and 38 patients, see Table 1 for mean scores). The results showed that affective alexithymia was negatively correlated to subclinical (CAPE) negative and depressive symptoms. Furthermore, the cognitive dimension was positively correlated to the CAPE negative symptoms (see Table 4). No significant correlations between the clinical symptoms (PANSS scores) and alexithymia dimensions were found (see Table 4).

**Table 4:** Correlations between the two alexithymia dimension and psychotic symptoms.

	Affective dimension	Cognitive dimension
*CAPE (n = 153)*		
Positive	*ρ* = -.20; *p* =. 01	*ρ* =. 07; *p* =. 37
Negative	*ρ* = -.26; *p* =. 001*	*ρ* =. 33; *p*<.001*
Depressive	*ρ* = -.34; *p*<.001*	*ρ* =. 15; *p* =. 08
*PANSS (n = 87)*		
Positive	*ρ* = -.06; *p* =. 56	*ρ* =. 08; *p* =. 47
Negative	*ρ* =. 17; *p* =. 13	*ρ* =. 05; *p* =. 64
General	*ρ* = -.17; *p* =. 12	*ρ* =. 18; *p* =. 11

*Significant at the corrected p<.008 (Bonferroni correction)

*Abbreviations*: CAPE: Community Assessment of Psychic Experiences; PANSS: Positive and Negative Syndrome Scale

The correlations between the BVAQ dimensions and the PANSS scores were repeated excluding all UHR subjects aged below 18. The correlations remained non-significant (see Table D in S2 File).

## Discussion

The aim of the current study was to examine alexithymia in patients with schizophrenia and subjects at increased risk for developing psychosis. The results revealed that siblings of patients with schizophrenia, individuals at UHR for psychosis and patients with schizophrenia show a type-II alexithymia pattern with higher cognitive alexithymia and equal or lower scores on the affective alexithymia dimension compared to controls. Furthermore, there appeared to be a parametric effect of risk on cognitive alexithymia scores with higher risk for schizophrenia being associated with more alexithymia: UHR subjects had higher cognitive alexithymia scores compared to siblings, who in turn scored higher than non-clinical controls. We also found that alexithymia was associated with negative and depressive symptoms in the controls and siblings, but not in the UHR individuals and patients with schizophrenia.

The current results support the idea that alexithymia might be part of the vulnerability for schizophrenia [7]. Patients with schizophrenia, as well as siblings and subjects at UHR for psychosis showed higher levels of cognitive alexithymia than non-clinical controls. These findings are in line with previous literature on patients with schizophrenia [7, 8, 11]. Furthermore, for the UHR group, these results corroborate the earlier published findings in a smaller group [4]. In siblings, van ‘t Wout et al. (2007) [7] showed a significant gender*group interaction on alexithymia scores, with male siblings showing higher scores on the verbalizing scale of the cognitive dimension compared to male controls. Our results did not show an interaction between group and gender on alexithymia scores, however.

In line with our hypothesis, the degree of risk for developing psychosis was associated with the cognitive alexithymia dimension. Subjects with an UHR for psychosis had higher scores on this dimension compared to siblings, who in turn scored higher than controls. Patients also differed from siblings on cognitive alexithymia, but only on the identifying subscale and not on other alexithymia subscales. This finding is agreement with the results of van ‘t Wout et al. (2007), who also showed that patients only differed from siblings on the identifying subscale. Our study goes beyond the study of van ‘t Wout et al. (2007) [7] by including larger samples and by including a comparison with UHR subjects. Notably, alexithymia scores did not differ between the UHR group and the patients. This pattern is in agreement with a previous study in which emotion recognition was more impaired in UHR individuals compared to relatives of patients with schizophrenia, while there were no significant differences on emotion recognition between the UHR and the patient group [37]. Furthermore, Amminger et al. (2012) also reported that UHR individuals and schizophrenia patients share the same emotion recognition problems [38]. Taken together, these results suggest that individuals at UHR for psychosis have similar cognitive emotional processing difficulties as patients. Furthermore, these difficulties are also present in siblings with a high genetic risk for psychosis, albeit to a lesser extent.

When examining the five subscales of alexithymia, underlying the two alexithymia dimensions, the results revealed that the UHR individiuals and patients with schizophrenia mainly differed from controls on the identifying and verbalizing subscales (although the difference between controls and patients on the verbalizing scale was not significant). This is in line with previous research [4, 7, 8, 11] and suggests that difficulties in identifying and verbalizing emotions might be specifically related to the vulnerability for psychosis. Experiencing these difficulties with identifiying and verbalizing emotions might result in emotional distress as was previously suggested by Fogley et al. (2014) [16] who revealed positive correlations between these alexithymia subscales and emotional distress in patients with schizophrenia. However, the sibling group did not differ from controls on identifying nor verbalizing. In fact, the largest difference between siblings and controls was found on the analyzing subscale, although not significant. Previous research has shown that siblings differed from controls on the verbalizing scale [7], which is contrast with the current findings. Therefore, further research is necessary to examine which particular alexithymia subscales are underlying the higher levels of cognitive alexithymia in this group.

The current results point to a type-II alexithymia pattern in patients as well as in the two high-risk groups. This type-II alexithymia pattern, with high scores on the cognitive dimension and normal or low scores on the affective dimension, has also previously been reported in patients with schizophrenia [7, 8], their siblings [7], and subjects at UHR for psychosis [4]. However, the current study is, to the best of our knowledge, the first study to directly compare these two high-risk groups. It has been suggested that especially this combination of alexithymia scores, awareness of emotional arousal without accompanying emotional cognition, may have negative consequences such as increased negative affect and anxiety [13, 14], which are also reported in patients with schizophrenia [39, 40]. Furthermore, this type-II alexithymia pattern appeared to be related to subclinical levels of negative and depressive symptoms. This finding is in line with a previous report in which a type-II alexithymia pattern was related to total schizotypy in a nonclinical sample [41]. Furthermore, positive associations between cognitive alexithymia and schizotypy have also been reported [42]. Remarkably, no significant associations between clinical symptoms and alexithymia were found in the UHR individuals and patients. Although some previous studies did report associations between symptoms and alexithymia in these groups [4, 7, 15], the majority of studies did not report any significant associations [9, 10, 16, 43, 44]. Furthermore, alexithymia does not appear to be related to psychotic symptoms in patients with schizophrenia over time [43]. It was previously indicated that although patients with schizophrenia often show aberrant levels of affective traits (e.g. neuroticism), these traits are relatively stable in these groups and not related to symptomatology during the phase of the illness [45]. However, in nonclinical samples, such as controls and relatives, these affective traits do appear to be related to symptomatology [45]. This in combination with the current findings suggests that a type-II alexithymia pattern might be specifically involved in the vulnerability for psychosis, rather than related to psychotic symptoms during the disorder itself. Moreover, a type-II alexithymia pattern might increase the vulnerability of developing psychosis. Future research should examine whether individuals with high levels of type-II alexithymia are indeed at higher risk for developing psychosis through longitudinal research. Furthermore, future studies should elucidate whether training or treatment in an early stage that targets the cognitive dimension of alexithymia, might be beneficial in attenuating subclinical symptoms in groups at high risk for psychosis.

Several limitations of this study should be addressed. First, the groups differed significantly on several demographic variables such as age, level of education and gender. These group differences are due to the fact that the groups are inherently difficult to match as patients with schizophrenia are more likely to be male than female [46] and subjects at UHR are younger because an age below 35 is one of the selection criteria to be considered UHR [26]. Moreover, subjects at UHR for psychosis and patients with schizophrenia are generally less educated than healthy controls. Controlling for these variables might have had a negative impact on the power of these analyses. However, we also performed the group analyses without controlling for these factors, which revealed almost the same pattern of differences on alexithymia scores between groups. Second, alexithymia was assessed through a self-report measure. Self-report measures rely to a certain extent on the ability to reflect on one’s own mental states, a capacity that might be compromised in individuals with alexithymia. Therefore, we recommend future studies to examine alexithymia with self-report and observer-rated measures. Third, previous research has indicated that cognition is related to alexithymia in patients with schizophrenia [16]. Although, previous research has shown that differences in alexithymia scores between controls and patients with schizophrenia, siblings or UHR individuals hold even after controlling for cognition [4,7], the lack of controlling for cognitive ability (except through the level of education) is a limitation of the current study. We therefore recommend future research to include cognitive measurements such as working memory or processing speed. Fourth, the two UHR samples (from Groningen and the sample from Van Rijn et al., (2007)) were included through different selection methods. Although these samples only differed on age and verbalizing, this might have increased the heterogeneity of the UHR sample, possibly resulting in lower power. Fifth, there is ongoing debate on whether the two dimensions of alexithymia exist [6, 32]. Therefore, future research should further examine the empirical support for the cognitive and affective dimension. Finally, the reliability of examining alexithymia in adolescents using the TAS-20 (a different alexithymia measure) is low and generally not recommended [35]. As far as we know, the reliability of the use of the BVAQ in an adolescents sample has not yet been investigated. Although the current results remained significant after excluding all subjects aged below 18, we recommend future research to examine the reliability of the BVAQ in adolescents.

In conclusion, these results indicate that the degree of risk for psychosis is related to higher levels of alexithymia. More specifically, groups at high risk for psychosis, as well as patients, show a type-II alexithymia profile with high levels of cognitive alexithymia and normal or slightly lower levels of affective alexithymia. Furthermore, alexithymia appeared to be related to nonclinical psychotic symptoms. These findings support the idea that alexithymia, especially the type-II pattern, might be a vulnerability factor for psychosis.

## Supporting Information

S1 File Supporting Tables. Table A, Test statistics of group differences on the alexithymia dimensions and subscales without including covariates. Table B, Post-hoc results (mean difference and p-value) of group differences on the cognitive and affective dimension without including covariates. * Significant at p<.05, corrected for multiple comparisons applying a Bonferroni correction *Abbreviations*: *HC*: *healthy controls; UHR*: *Ultra-High Risk*. Table C, Post-hoc results (mean difference and p-value) of group differences on the cognitive alexithymia subscales and the fantasizing subscale without including covariates. * Significant at p<.05, corrected for multiple comparisons applying a Bonferroni correction *Abbreviations*: *HC*: *healthy controls; UHR*: *Ultra-High Risk*
(DOCX)

S2 File Supporting Tables. Table A, Test statistics of group differences on the alexithymia dimensions and subscales excluding subjects aged below 18. Table B, Post-hoc results (mean difference and p-value) of group differences on the cognitive and affective dimension excluding subjects aged below 18. * Significant at p<.05, corrected for multiple comparisons applying a Bonferroni correction *Abbreviations*: *HC*: *healthy controls; UHR*: *Ultra-High Risk*. Table C, Post-hoc results (mean difference and p-value) of group differences on the cognitive alexithymia subscales excluding subjects aged below 18. * Significant at p<.05, corrected for multiple comparisons applying a Bonferroni correction *Abbreviations*: *HC*: *healthy controls; UHR*: *Ultra-High Risk*. Table D, Correlations between the two alexithymia dimension and psychotic symptoms excluding subjects aged below 18. *Significant at the corrected p<.008 (Bonferroni correction); *Abbreviations*: PANSS: Positive and Negative; Syndrome Scale
(DOCX)

S3 File Supplemental Data File. (SAV)
