# Supplementary material for: Cognitive Alexithymia Is Associated with the Degree of Risk for Psychosis
Source: PLoS One. 2015 Jun 1;10(6):e0124803. doi: 10.1371/journal.pone.0124803 (PMC4451258; doi:10.1371/journal.pone.0124803)
Supplement: S1 File — Table A, Test statistics of group differences on the alexithymia dimensions and subscales without including covariates. Table B, Post-hoc results (mean difference and p-value) of group differences on the cognitive and affective dimension without including covariates. * Significant at p<.05, corrected for multiple comparisons applying a Bonferroni correction Abbreviations: HC: healthy controls; UHR: Ultra-High Risk. Table C, Post-hoc results (mean difference and p-value) of group differences on the cognitive alexithymia subscales and the fantasizing subscale without including covariates. * Significant at p<.05, corrected for multiple comparisons applying a Bonferroni correction Abbreviations: HC: healthy controls; UHR: Ultra-High Risk (DOCX) [file pone.0124803.s001.docx]

**Supplement 1**

*These tables represent the results of the (M)ANOVA’s without including gender as a factor and without age and education as covariates.*

**Table A**

Test statistics of group differences on the alexithymia dimensions and subscales without including covariates.

|  |  |
| --- | --- |
|  | Test statistic |
| Cognitive dimension | F_3,289_=23.8; p<.001 |
| Verbalizing | F_3,289_=12.0, p<.001 |
| Identifying | F_3,289_=31.8, p<.001 |
| Analyzing | F_3,289_=6.2 p<.001 |
| Affective dimension | F_3,289_=2.7; p=.04 |
| Fantasizing | F_3,289_=4.2, p=.006 |
| Emotionalizing | F_3,289_=.08, p=.97 |

**Table B**

Post-hoc results (mean difference and p-value) of group differences on the cognitive and affective dimension without including covariates

|  |  | Cognitive dimension | Affective dimension |
| --- | --- | --- | --- |
| HC | Siblings | -6.5; p=.01* | 1.0; p=1.0 |
|  | UHR | -19.2; p<.001* | 4.9; p=.03* |
|  | Patients | -15.9; p<.001* | 2.0; p=1.0 |
| Siblings | UHR | -12.8; p<.001* | 3.9; p=.19 |
|  | Patients | -9.4; p=.008* | .95; p=1.0 |
| UHR | Patients | 3.4; p=1.0 | -2.9; p=1.0 |

* Significant at p<.05, corrected for multiple comparisons applying a Bonferroni correction
*Abbreviations: HC: healthy controls; UHR: Ultra-High Risk*

**Table C**

Post-hoc results (mean difference and p-value) of group differences on the cognitive alexithymia subscales and the fantasizing subscale without including covariates

|  |  | Verbalizing | Identifying | Analyzing | Fantasizing |
| --- | --- | --- | --- | --- | --- |
| HC | Siblings | -1.9; p=.40 | -.88; p=1.0 | -1.7; p=.22 | -.66; p=1.0 |
|  | UHR | -7.0; p<.001* | -7.5; p<.001* | -3.4; p=.004* | 3.5; p=.02* |
|  | Patients | -4.1; p=.02* | -6.5; p<.001* | -3.8; p=.004* | .65; p=1.0 |
| Siblings | UHR | -5.1; p<.001* | -6.6; p<.001* | -1.7; p=.55 | 4.2; p=.004* |
|  | Patients | -2.3; p=.61 | -5.6; p<.001* | -2.1; p=.36 | 1.3; p=1.0 |
| UHR | Patients | 2.9; p=.39 | .95; p=1.0 | -.42; p=1.0 | -2.9; p=.35 |

* Significant at p<.05, corrected for multiple comparisons applying a Bonferroni correction
*Abbreviations: HC: healthy controls; UHR: Ultra-High Risk*
