# Supplementary material for: Cognitive Alexithymia Is Associated with the Degree of Risk for Psychosis
Source: PLoS One. 2015 Jun 1;10(6):e0124803. doi: 10.1371/journal.pone.0124803 (PMC4451258; doi:10.1371/journal.pone.0124803)
Supplement: S2 File — Table A, Test statistics of group differences on the alexithymia dimensions and subscales excluding subjects aged below 18. Table B, Post-hoc results (mean difference and p-value) of group differences on the cognitive and affective dimension excluding subjects aged below 18. * Significant at p<.05, corrected for multiple comparisons applying a Bonferroni correction Abbreviations: HC: healthy controls; UHR: Ultra-High Risk. Table C, Post-hoc results (mean difference and p-value) of group differences on the cognitive alexithymia subscales excluding subjects aged below 18. * Significant at p<.05, corrected for multiple comparisons applying a Bonferroni correction Abbreviations: HC: healthy controls; UHR: Ultra-High Risk. Table D, Correlations between the two alexithymia dimension and psychotic symptoms excluding subjects aged below 18. *Significant at the corrected p<.008 (Bonferroni correction); Abbreviations: PANSS: Positive and Negative; Syndrome Scale (DOCX) [file pone.0124803.s002.docx]

**Supplement 2**

*These tables represent the results excluding all subjects aged below 18. Excluding these subjects resulted in 22 UHR individuals (the number of controls, siblings and patients remained the same).*

**Table A**

Test statistics of group differences on the alexithymia dimensions and subscales excluding subjects aged below 18.

|  |  |
| --- | --- |
|  | Test statistic |
| Cognitive dimension | F_3,253_=15.9; p<.001 |
| Verbalizing | F_3,253_=11.0, p<.001 |
| Identifying | F_3,253_=16.8, p<.001 |
| Analyzing | F_3,253_=3.2 p=.02 |
| Affective dimension | F_3,253_=3.2; p=.04 |
| Fantasizing | F_3,253_=1.6, p=.19 |
| Emotionalizing | F_3,253_=1.1, p=.35 |

**Table B**

Post-hoc results (mean difference and p-value) of group differences on the cognitive and affective dimension excluding subjects aged below 18

|  |  | Cognitive dimension | Affective dimension |
| --- | --- | --- | --- |
| HC | Siblings | -7.0; p=.002* | .88; p=1.0 |
|  | UHR | -21.5; p<.001* | 7.6; p=.02* |
|  | Patients | -13.3; p<.001* | 3.3; p=.79 |
| Siblings | UHR | -14.5; p<.001* | 6.7; p=.07 |
|  | Patients | -6.3; p=.26 | 2.4; p=1.0 |
| UHR | Patients | 8.2; p=.34 | -4.3; p=.99 |

* Significant at p<.05, corrected for multiple comparisons applying a Bonferroni correction
*Abbreviations: HC: healthy controls; UHR: Ultra-High Risk*

**Table C**

Post-hoc results (mean difference and p-value) of group differences on the cognitive alexithymia subscales excluding subjects aged below 18

|  |  | Verbalizing | Identifying | Analyzing |
| --- | --- | --- | --- | --- |
| HC | Siblings | -2.1; p=.17 | -1.0; p=1.0 | -1.9; p=.07 |
|  | UHR | -9.8; p<.001* | -7.8; p<.001* | -1.6; p=1.0 |
|  | Patients | -3.6; p=.10 | -5.5; p<.001* | -2.9; p=.09 |
| Siblings | UHR | -7.7; p<.001* | -6.8; p<.001* | .28; p=1.0 |
|  | Patients | -1.5; p=1.0 | -4.5; p=.001* | -.96; p=1.0 |
| UHR | Patients | 6.2; p=.02* | 2.4; p=.82 | -1.2; p=1.0 |

* Significant at p<.05, corrected for multiple comparisons applying a Bonferroni correction
*Abbreviations: HC: healthy controls; UHR: Ultra-High Risk*

**Table D**

Correlations between the two alexithymia dimension and psychotic symptoms excluding subjects aged below 18

|  | Affective dimension | Cognitive dimension |
| --- | --- | --- |
| *PANSS (n=59)* |  |  |
| Positive | *ρ*=.23; *p*=.09 | *ρ*=.02; *p*=.86 |
| Negative | *ρ*=.08; *p*=.56 | *ρ*=-.07; *p*=.59 |
| General | *ρ*=.15; *p*=.26 | *ρ*=.08; *p*=.54 |

*Significant at the corrected p<.008 (Bonferroni correction); *Abbreviations:* PANSS: Positive and Negative Syndrome Scale
